# Catch, Modify and Analyze: Methods of Chemoselective Modification of Cysteine-Containing Peptides

**DOI:** 10.3390/molecules27051601

**Published:** 2022-02-28

**Authors:** Marta Kowalska, Remigiusz Bąchor

**Affiliations:** Faculty of Chemistry, University of Wroclaw, 50-383 Wroclaw, Poland; marta.kowalska@chem.uni.wroc.pl

**Keywords:** cysteine, cysteine-containing peptide, chemoselective modification, peptide enrichment, charge derivatization, Michael addition

## Abstract

One effective solution in the analysis of complex mixtures, including protein or cell hydrolysates, is based on chemoselective derivatization of a selected group of compounds by using selective tags to facilitate detection. Another method is based on the capture of the desired compounds by properly designed solid supports, resulting in sample enrichment. Cysteine is one of the rarest amino acids, but at least one cysteine residue is present in more than 91% of human proteins, which clearly confirms its important role in biological systems. Some cysteine-containing peptides may serve as significant molecular biomarkers, which may emerge as key indices in the management of patients with particular diseases. In the current review, we describe recent advances in the development of cysteine-containing peptide modification techniques based on solution and solid phase derivatization and enrichment strategies.

## 1. Introduction

Investigation of peptide and protein biomarkers of cellular and tissue proteomes is limited, due to the complex nature of the samples. Therefore, the development of enrichment methods for some groups of peptides, based on chemoselective fractionation or derivatization is an important task which may facilitate proteomics studies [1,2].

Cysteine is an endogenous amino acid which, next to methionine and homocysteine, belongs to the group of sulfur containing amino acids [3]. The cysteine residue plays an important role in the structure of proteins. Among other things, it is responsible for binding metal ions and holding them in place. In proteins, the cysteine side chain (sulfhydryl) functional group can be easily oxidized, leading to the formation of a disulfide bridge between the two cysteines, which strengthens the tertiary and quaternary structures of the protein. The presence of disulfide bridges makes proteins more resistant to thermal denaturation, thus maintaining their activity over a wider temperature range [4]. At least one cysteine residue is present in 91% of known human proteins [1], making this residue a significant target for chemoselective fractionation. Moreover, the thiol group of cysteine shows high reactivity—it is active redox and has strong nucleophilic properties (the most nucleophilic group among the functional groups of amino acids [5])—resulting from the large atomic radius of sulfur and the low dissociation energy of the thiol bond [6]. Strong nucleophilic properties make cysteine easily modified by electrophilic and thiol-disulfide reagents.

Several strategies of thiol-selective modification of cysteine-containing proteins have been reported previously [7,8,9]. Chalker and co-workers [7], highlighted the utility of the cysteine residue in protein modification with particular attention to chemical reactions that lead to a better understanding of natural protein modification and macromolecular function. In 2016 Gunnoo and Madder [8], presented another review focused on methods involving cysteine as a precursor for chemical modification of proteins since 2009, when Chalker et al. [7] reviewed the topic. Recently, an interesting and valuable review describing new reagents that allow for efficient cysteine-containing compound conjugation in solution, leading to products with significant pharmaceutical application, was presented by Ochtropp and Hackenberger [9]. In this manuscript, the ongoing effort to develop new thiol-selective reagents, including metal-based reagents, substituted electrophilic reagents, and unsaturated electrophiles, characterized by higher labeling efficiencies and improved stabilities were described. Additionally, Ochtropp and Hackenberger reported the possibility of the formation of new therapeutics, including antibody-drug conjugates, in the form of cysteine-conjugated derivatives. 

Due to the importance of cysteine-containing peptides, resulting from their role in biological systems, different strategies for their enrichment (catching) and modification have been developed [10,11,12,13,14]. In general, methods of cysteine-containing peptide enrichment can be divided into enrichment in solution, or on solid supports. In this review we concentrate on cysteine-containing peptide enrichment by thia-Michael reaction (Michael addition) [15,16], thiol-disulfide exchange [17] and charge derivatization [18].

## 2. Cysteine-Containing Peptide Enrichment in Solution

### 2.1. Michael Addition

**Figure 1:**
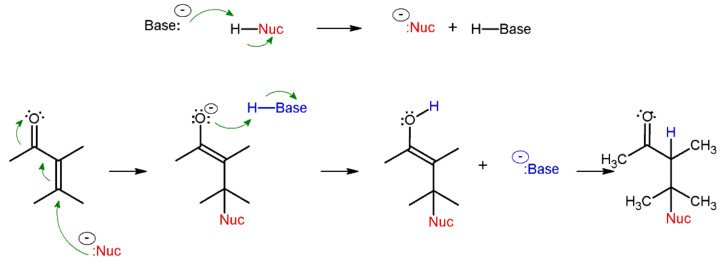
Schematic representation of the Michael addition reaction mechanism.

The Michael addition reaction is an enolate type nucleophile reaction to α, β-unsaturated carbonyl moiety containing compounds. This reaction type is base-catalyzed and occurs in the presence of a nucleophile, resulting in the formation of an anti-Markovnikov addition product (Figure 1), such as in the thiol-ene addition formed according to a radical mechanism [19]. The thiol-Michael reaction can be affected by the thiol’s basicity, the strength of the base or the polarity of the solvent. Additionally, the reaction rate is influenced by the steric hindrance of the reactants; the presence of larger substituents in the α and β positions on the Michael acceptor can hamper the reaction [20,21]. Moreover, a high steric hindrance around the nucleophilic center decreases its reactivity to the Michael acceptor [22].

**Figure 2:**
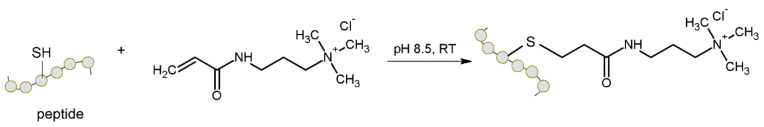
The reaction of 3-(acrylamidopropyl)trimethylammonium chloride with a cysteine residue in a peptide [23].

In 2004 Ren et al. [23] developed a simple strategy for specifically targeting cysteine-containing peptides in a tryptic digest. The method uses the quaternary amine tag (QAT) to perform charge derivatization, which facilitates the qualitative and quantitative analysis by mass spectrometry, allowing for the ultrasensitive detection of modified compounds. First, reduction of the disulfide bonds was performed, and then tags were introduced by derivatizing cysteine residues with (3-acrylamidopropyl)trimethylammonium chloride (Figure 2). Derivatized cysteine-containing peptides were enriched by strong cation exchange chromatography. The use of QAT not only increases the ionization efficiency of cysteine-containing peptides, but also enables chromatographic selection with simple cation exchange chromatography columns.

The developed method allowed for the identification of 28 peptides containing cysteine from 33 peptides obtained after trypsin digestion. Four of the five non-specifically linked peptides contained a histidine residue. Due to the small number of non-specifically bound peptides, no further purification steps were used. However, it should be noted that this strategy is charge dependent, and therefore peptides containing multiple glutamic acid or aspartic acid residues will be negatively charged and will not be captured by the column. The presented studies have shown that the use of a quaternary amine tag in combination with strong cation exchange chromatography enables a high degree of selectivity in the capture of cysteine-containing peptides. The presence of QAT additionally increases the ionization efficiency during mass spectrometry analysis, which increases the possibility of reliable compound identification [20]. 

The most commonly used electrophilic reagent for modification of cysteine residues is maleimide and its derivatives [24], due to the high selectivity and irreversibility of the maleimide reaction with the cysteine residue [25,26,27]. Modification of the maleimide and introduction of additional functional groups may further increase the number of biomolecules that could be subsequently modified. For example, maleimide converting into bromomaleimide gave three sites to which biological compounds could attach to form bioconjugates. These sites are two carbon atoms involved in the formation of a double bond and a nucleophilic nitrogen atom, and the reaction of maleimide with thiol groups of peptides or proteins is reversible [28]. Dibromomaleimide and ethyl maleimide can also be used for selective reactions with cysteine residues [5].

**Figure 3:**
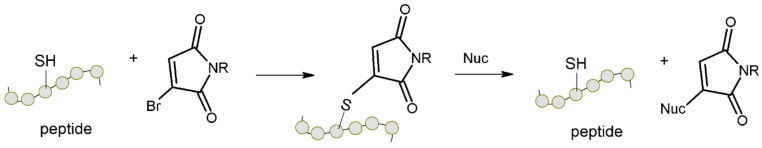
Reaction scheme of a cysteine residue with a bromomaleimide, and the reversal reaction caused by a nucleophile (Nuc) [25].

In 2009 Tedaldi et al. [25] studied bromomailimides as new reagents for the selective and reversible modification of cysteine. They demonstrated that incorporation of a leaving group on the maleimide double bond enables an addition–elimination sequence to occur on reaction with cysteine. The received addition reaction product was a thiomaleimide, which retains a double bond. This thiomaleimide has a new reactivity profile that allowed for its use in subsequent steps. A nucleophile can add to thiomaleimide in another conjugate addition to C-3 and cleave the cysteine, reversing the modification (Figure 3).

It has been demonstrated that bromomaleimides react selectively and rapidly with cysteine residues. They can be used for reversible cysteine modification, as the thiomaleimide conjugate can be cleaved with dithiothreitol (DTT) or tris(2-carboxyethyl)phosphine) [25].

### 2.2. Thiol-Disulfide Exchange

**Figure 4:**
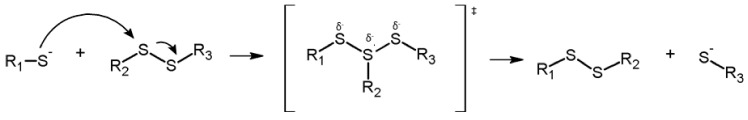
Schematic representation of a thiol-disulfide exchange mechanism.

Thiols can be oxidized to disulfides and disulfides can be reduced to thiols. Although the products of oxidation or reduction reactions are often the same as those of thiol-disulfide exchange reactions, the pathways are mechanistically different [29,30]. The thiol-disulfide exchange reaction proceeds according to a S_N_2 reaction mechanism. The nucleophilic thiolate anion (RS-) attacks one of the sulfur atoms in the disulfide, leading to the formation of a new disulfide bond and the simultaneous release of a new thiolate (Figure 4). The thiol-disulfide exchange reactions are biologically important—they play a significant role in many aspects of cellular function. These reactions stabilize the protein structure and may rearrange upon conformational changes of protein, due to the chemically labile and dynamic nature of disulfide bonds [31].

**Figure 5:**
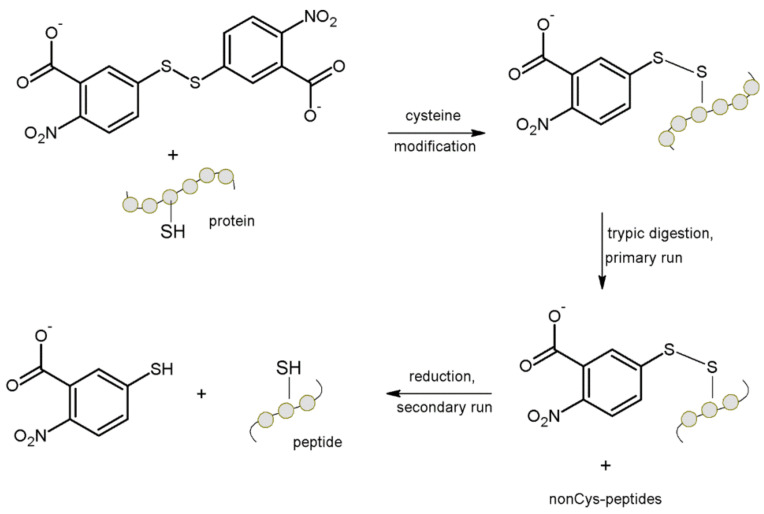
Reactions employed for the isolation of cysteine-containing peptides [11].

In 2004 Gevaert et al. [11], proposed a procedure for specific isolation of cysteine-containing peptides from a complex peptide mixture. The procedure consisted of mixing cysteine with Ellman’s reagent, disulfide formation, which caused conversion of cysteine to hydrophobic residues. Proteins were then digested with trypsin and reverse-phase high-performance liquid chromatography was used to fractionate the generated peptide mixture. Cysteine-containing peptides were isolated from each primary fraction by a reduction step, followed by a secondary peptide separation on the same column. Conditions for the second separation were identical to the primary separation (Figure 5). 

The reducing agent makes cysteine-peptides more hydrophilic by removing the covalently attached group from the side chain. Such peptides can be selectively collected during the secondary separation, and can be used to identify their final precursor proteins by automated LC-MS/MS. 

The reported method was applied to the analysis of the human platelet proteome and enriched human plasma. In both proteomes used, extremely abundant proteins and a significant number of low abundance proteins were identified. A range for protein identification spanning 4–5 orders of magnitude was demonstrated.

Many proteins were identified in the conducted study, and more than 50% of them were identified by cysteine-containing peptide capture. Additionally, the contaminating non-cysteine containing peptides seemed to be mainly attributed to the major plasma proteins in the sample, which were also identified in a single LC-MS/MS run without cysteine-peptide enrichment. Although such peptides were present in the final mixture, they did not affect the LC-MS/MS analysis. [11]

#### Application of Nanoparticles

Another strategy for capturing peptides is based on the nanoparticles application. The application of nanosized materials to proteomics research provides many immediate advantages, such as higher specificity, faster binding rates, higher surface-to-volume areas and higher miscibility [32]. In 2004 Xu et al. [33] developed a new specific method of histidine-tagged proteins magnetic separation with use of nitrilotriacetic acid-immobilized superparamagnetic iron oxide. To connect nitrilotriacetic acid to the iron oxide shell of magnetic nanoparticles the dopamine was used. In this method, the surfaces of silica NPs are readily modified to present a numerous of functional groups. In 2007 Guo et al. [34] proposed a technique for a soluble polymer-based isotopic labelling and specific, efficient capturing of cysteine-containing peptides with use of functionalized dendrimers. 

**Figure 6:**
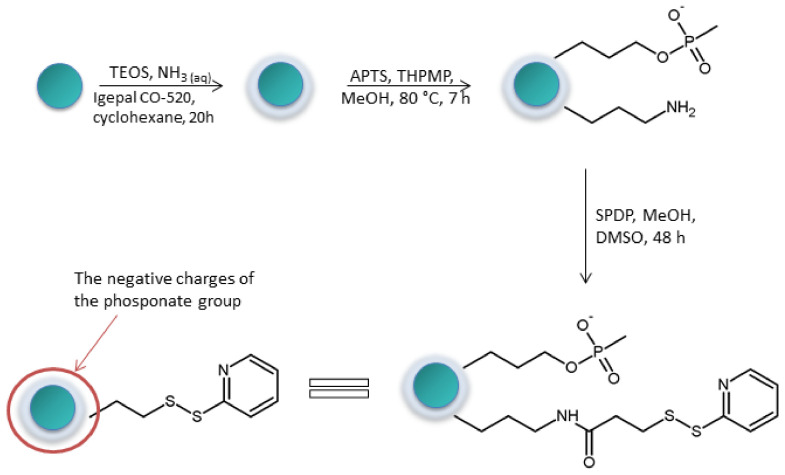
Schematic presentation of Fe_3_O_4_@SiO_2_ core shell nanoparticles’ modification. TEOS—tetraethylorthosilicate, Igepal—nonyl phenol ethoxylate, APTS—Aminopropyltriethoxysilane, THPMP—3-(Trihydroxysliyl)propylmethylphosphonate, SPDP—*N*-Succinimidyl 3-(2-Pyridyldithio)-propionate, DMSO—dimethyl sulfoxide [35].

Another method was developed by Palani and co-workes in 2008 [35]. The proposed technique was based on the application of superparamagnetic Fe_3_O_4_@SiO_2_ core-shell nanoparticles (ca. 30 nm diameter) (Figure 6). Their surface have been modified with a thiol-specific functional group and showed high efficiency of capturing of cysteine-containing peptides without contamination from other, non-specifically interacting peptides. The authors unambiguously confirmed the specificity and the efficiency of the cysteinyl proteome isolation by LC/MS/MS analysis. The proposed method was based on the protocol developed previously by Liu et al. [36] (the description is provided later in the paper). However, Palani and co-workers [32] demonstrated how this protocol can be useful as proteomic technique for more complex proteome samples. 

The functionalized magnetic nanoparticles are promising materials for proteome fractionation and the enrichment of low-abundance proteins without complications caused by nonspecific proteins, which is an important feature for the effective discovery of biomarkers or disease target molecules [35].

### 2.3. Charge Derivatization and Mas Tagging

For weakly ionizing compounds, whose analysis by mass spectrometry is difficult or impossible, a good solution may be a modification that creates or introduces a group containing a positive or negative charge. For such derivatization to be feasible, it is imperative that the test compound possesses a functional group capable of reacting with the reagent used to carry out the charge modification [37]. The introduced or created ionization tag has a permanent charge that does not dissociate in solution, which most often results from the chemical structure of the compound used for derivatization. It can be, for example, a group containing a quaternary nitrogen or phosphorus atom or a tertiary sulfur atom [38].

Not all proteins and peptides are easily ionized. Several functional groups that can be derivatized easily are present in the peptide structure. These are amino groups at the *N*-terminus of the peptide chain, or on the lysine side chain, or a carboxyl group at the *C*-terminus of the peptide, or on the glutamic and aspartic acid side chains [39,40], as well as a thiol group on a cysteine residue. It is also possible to target functional groups that occur as post-translational modifications, such as phosphorylation [41], and glycosylation [42,43,44]. 

Charge derivatization of the amine group has been known for many years. One of the first proposals to modify the amino group was to use a reaction with methyl iodide, in which the amino group was methylated three times, leading to the formation of a quaternary ammonium salt [45]. However, the derivatization efficiency was low and characterized by low specificity, therefore new modification methods were still sought and developed. An example was the use of diazomethane as an amino group derivatizing reagent in peptides immobilized on an ion chromatography column (involved in cation exchange), resulting in *N*,*N*,*N*-trimethylated peptides [46]. Both the *N*-terminus of the peptide and the amino group of the lysine residue and the imidazole groups, can be modified by this method. Unfortunately, carboxyl groups and tyrosine also react, although no positive charge is formed. 

**Figure 7:**
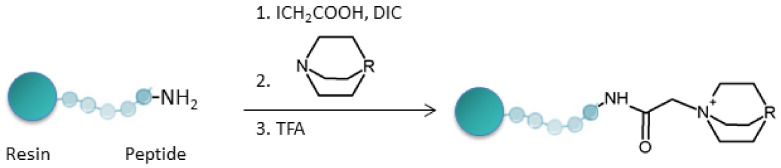
Schematic representation of quaternary ammonium salt formation at the amino group of the *N*-terminus of a peptide with DABCO. DIC—*N*,*N*’-diisopropylcarbodiimide [47].

The use of ionization markers, which are higher betaine analogs, allows an increase in the sensitivity of the ESI-MS analysis by reducing the peptide detection threshold to the level of sub-femtomoles or attomoles in MRM mode (Multiple Reaction Monitoring) [47]. However, it has been shown that betaine derivatives undergo Hoffman elimination during the MS/MS experiment, which complicates the analysis and interpretation of the mass spectra. A solution to this problem was proposed by Setner et al. [48]. Charge derivatization was based on bicyclic amines with a stable, rigid structure, such as 1-azabicyclo[2.2.2]octane (ABCO) or 1,4-diazabicyclo[2.2.2]octane (DABCO). The modification was also carried out on a solid support (Figure 7). We successfully used solid-support derivatization to analyze the OBOC (one-bead-one-compound) peptide library by MS/MS analysis of a trace amount of a compound (femtomolar) obtained from single resin grains [49,50]. 

Other types of peptide ionization tags described previously by us [51] are the 2,4,6-trimethylpyryl and 2,4,6-triphenylpyryl salts, such as triphenylpyryl tetrafluoroborate (TPP), which allow the modification of the α-amino groups of glycine and alanine, as well as the ε-amino group of the lysine residue. Derivatization reagents are inexpensive and can be easily prepared by cyclization, by reacting benzoacetophenone and benzaldehyde (2:1 molar ratio) [52]. The derivatization reaction is carried out in the presence of *N*,*N*,*N*-triethylamine at 60 °C in DMF. Such a modification made it possible to analyze attomolar amounts of the model peptide using the MRM method. In our previous studies we used LC-MS-MRM method in the analysis of podocin (podocyturia biomarker) in tryptic digests of feline [53], canine [54], horse [55] and human urine samples [56]. We also proposed a technique to increase sensitivity and detectability of tryptic peptides—a charge derivatization with a quaternary ammonium tag in a the form of the 2,4,6-triphenypyrylium salt [44,48,57]. Furthermore, we compared and presented the advantage of the LC-MS method over the ELISA test in clinical diagnoses requiring identification of podocin [58]. 

**Figure 8:**
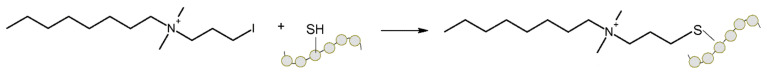
Scheme of selective modification of a cysteine residue with *N*-(3-iodopropyl)-*N*,*N*,*N*-dimethyloctylammonium iodide [59].

Modifications of the cysteine residue have the advantage that they concern the most nucleophilic functional group found in peptides, -SH, and thus the reaction can proceed selectively [59]. This amino acid is rare in protein sequences, therefore the targeted analysis of thiopeptides significantly reduces the complexity of the peptide mixture. The thiol group is an excellent nucleophile, it easily undergoes an addition reaction to an acrylamide group and is also able to form sulfides in a S_N_2 substitution reaction with an alkyl halide in a basic environment. In the case of using *N*-(3-iodopropyl)-*N*,*N*,*N*-dimethyloctylammonium iodide as a derivatizing reagent (Figure 8), using peptides derived from human growth factor, the signal was amplified 5–6 times on the MS spectrum, which means that the detection threshold dropped significantly [60]. On the other hand, the reaction of the thiopeptide with 1-[3-[(2-iodo-1-oxyethyl)amino]propyl]-3-butylimidazole bromide turned out to be very efficient and runs at 100% [34]. 

**Figure 9:**
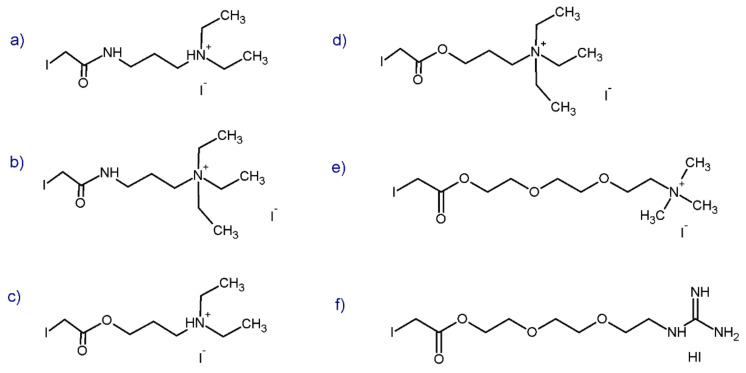
Structure of the cysteine tags proposed by Shimada and co-workers presenting amide-linked tags (**a**,**b**), ester-linked tags based on the tertiary amine moiety (**c**), other quaternary ammonium tags in ester forms (**d**,**e**) and guanidine tag (**f**) [61].

In 2012 Shimada et al. [61] developed and characterized six new cysteine mass tags for peptide enrichment and analysis (Figure 9). The tags were designed to contain a thiol-reactive group (iodacetyl) and have a hydrophilic character to reduce sample loss. Additionally, a tertiary amino group, a quaternary amino group, or a guanidino group were introduced to increase the proton affinity. The designed tags did not have an amide bond which minimized tag fragmentation during collision-induced dissociation.

In comparison with the iodoacetamide tag, the received tags caused 2- to 200-fold enhancement in sensitivity of the analyzed peptides. Amide-linked tags (Figure 9a,b) showed far more fragmentation at the linkage than analogous ester-linked tags (Figure 9c,d). In the case of tags with a quaternary moiety (Figure 9b,d) and tertiary amine moiety, the first group of tags gave better results. In a comparison of quaternary ammonium (Figure 9e) and guanidine (Figure 9f) tags, a better enhancing effect was observed with the former quaternary ammonium tag (Figure 9e). Among tested tags, the TM-DEG-IA, 8-iodoacetoxy-3,6-dioxaoctyltrimethylammonium iodide tag (Figure 9e) showed optimal sensitivity-enhancing effects. However, all designed tags can be used for MS-based analyzes of hydrophobic peptides and low-abundance biomarkers. In the case of biomarker quantitation by MS, cysteine-containing peptides can be important candidates because of the stable ion generation by the derivatization of designed mass tags [58,62].

#### 2.3.1. ICAT Strategy

**Figure 10:**
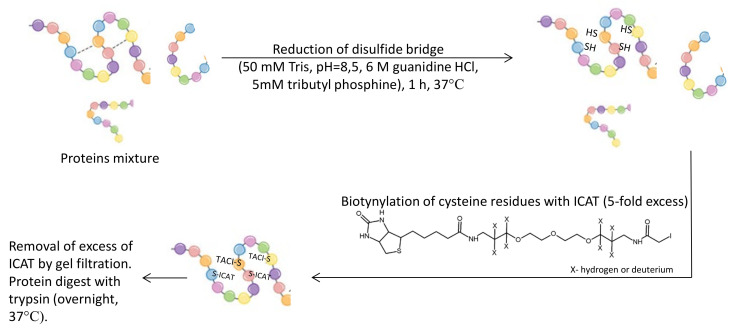
Schematic representation of cysteine residue tagging in protein samples. Tris—tris(hydroxymethyl)aminomethane, ICAT—isotope-coded affinity tags [63].

In 1999 Gygi et al. [63] described a method for the accurate quantification and simultaneous sequencing of individual proteins in a complex mixture. The method was based on isotope-coded affinity tags (ICAT) and tandem mass spectrometry analysis. The ICAT method was based on stable isotope labeling of proteins after isolation. The procedure includes reduction of disulfide bridges, biotinylation of cysteine residues with ICAT, and protein digest by trypsin, (Figure 10). The approach was applied to compare protein expression in the yeast *Saccharomyces cerevisiae*, using either ethanol or galactose as a carbon source. Results of this research illustrated the potential of the ICAT method for the identification of protein components and quantitative analysis. Cysteine-containing peptides were selectively isolated, which proved that the ICAT method reduced the complexity of the peptide mixture. 

During 2D gel analysis, only highly abundant proteins can be measured, when total cell lysates are applied [64,65]. The general reason is that the quantities of protein that can be loaded onto an analytical 2D gel are only in high microgram amounts. Using the ICAT strategy, any amount of starting material can be used and sufficient amounts of very low-abundance proteins can be prepared and detected by mass spectrometry. Additionally, the ICAT approach provides a broadly applicable means to compare quantitatively, global protein expression in cells and tissue in a variety of normal, developmental, and disease states. Furthermore, the proposed method can be extended to include reactivity toward other functional groups. ICAT reagents with different specificities could also make cysteine-free proteins susceptible to analysis by the ICAT method [58].

#### 2.3.2. Mass Tagging

**Figure 11:**
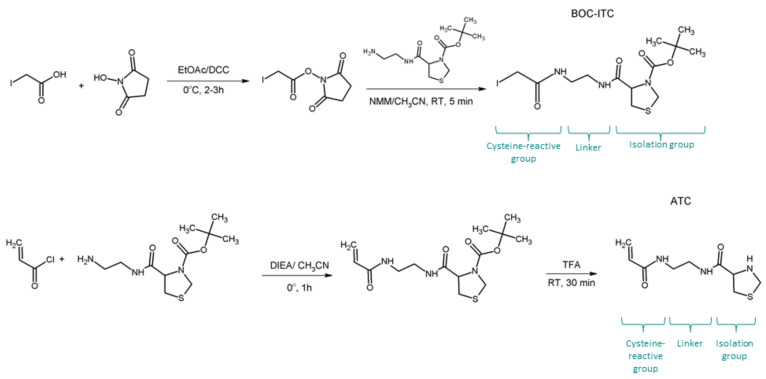
Chemical synthesis of the Boc-ITC and ATC. EtOAc—ethyl acetate, DCC—*N,N*′-dicyclohexylcarbodiimide, NMM—*N*-methylmorpholine, TFA—trifluoroacetic acid, DIEA—*N,N*-diisopropylethylamine, RT- room temperature [66].

In 2009 Giron et al. [66], demonstrated the benefits of the covalent capture (CC) method to enrich *N*-terminal cysteine-containing peptides, both in silico and in proof of principle experiments. In the same year, the authors extended previous strategies [67]. They described the synthesis and application of two new cysteinyl tags—cysteine-reactive covalent capture tags (C3T), for the capture of cysteine-containing peptides (Figure 11). These tags react specifically with cysteine through iodoacetyl and acryloyl moieties, which allow efficient and selective capture of cysteine-containing peptides by the covalent capture method. The thioproline group has been chosen as an isolating group and after a deprotection/activation step, a thiazolidine has been formed with an aldehyde resin. The coupling between aldehydes and an *N*-terminal Cys included in a tag is very specific and stable over a wide pH range (from 4 to 8). The *N*-[2-((2-acryloyl)amino)ethyl]-1,3-thiazolidine-4-carboxamide (ATC) tag was shown to provide the best results with capture of almost all theoretically expected peptides. It did not induce self-alkylation or side products that disturb the MS analysis, unlike tert-butoxycarbonyl-*N*-[2-((2-iodoacetyl)amino)-ethyl]-1,3-thiazolidine-4-carboxamide (Boc-ITC) tag [62].

The applicability of the enrichment strategy was demonstrated on small synthetic peptides and subsequently on peptides derived from digested proteins. Combining CC and C3T allow the observation of significant increases in protein coverage, reduces sample complexity and accesses low abundance proteins. The proposed method showed 100% specificity in tagging and enrichment of cysteine-containing peptides. MS and MS/MS analysis confirmed the efficient and straightforward selection of the cysteine-containing peptides. The synthesized tags do not interfere with peptide fragmentation. Furthermore, after covalent capture and release, the tagged peptides carry a free *N*-terminal cysteine residue that can easily undergo further modification, to enhance ionization efficiency for MS analysis [20,62,68]. 

#### 2.3.3. Isotopic Labeling

**Figure 12:**
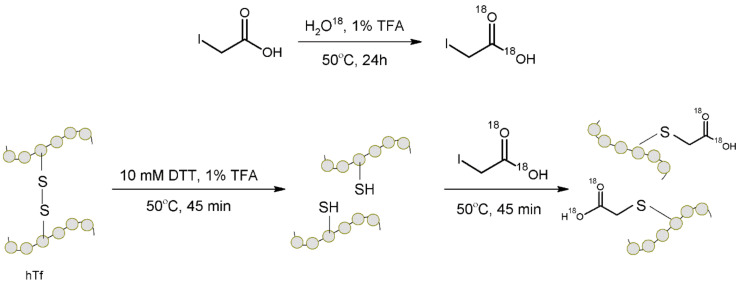
Representation of synthesis of labeled iodoacetic acid, and reaction of this labeled tag with thiol groups of cysteine residues. TFA—trifluoroacetic acid, DTT—dithiothreitol [69].

In 2012 Wang et al. [69] developed a new ^18^O labeling protocol for quantitation of cysteine-containing proteins using LC/MS. In the presented strategy, labeling was performed during the reduction or alkylation of the side chains of the cysteine residues with ^18^O-labeled iodoacetic acid prior to protein digestion. Labeling prior to protein digestion makes the quantification results peptide-independent. Labeled iodoacetic acid was prepared by isotope exchange of carboxylic oxygen atoms conducted in water enriched with ^18^O at an acidic pH. The developed marker was characterized by high stability at a mild pH, which demonstrated the required robustness in the sample processing stages. The advantage of this method is the high efficiency of incorporation of ^18^O into peptides. Furthermore, the combination of unlabeled and labeled samples at the protein level excludes all sources of quantitative errors that may occur during the introduction of the label at the level of the digested protein. The developed method was used to modify cysteine residues in human serum transferrin (hTf) (Figure 12). However, the proposed approach may also be suitable for biopharmaceutical analyses (pharmacokinetic studies, quality control of protein therapeutics [69]. 

**Figure 13:**
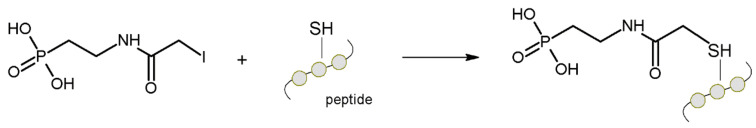
Schematic representation of cysteine-containing peptides labeling with CysPAT tag [70].

In 2016 Huang and co-workers [70] described another strategy of capturing cysteine-containing peptides. They synthesized a cysteine-specific phosphonate adaptable tag (CysPAT) to selectively label cysteine-containing peptides. The CysPAT was synthesized with use of N-Succinimidyl iodoacetate (SIA) and 2-aminoethylphosphonic acid (2-AEP). The synthesized tag was used to selectively label Cys peptides (Figure 13) followed by enrichment with TiO_2_ and subsequent mass spectrometric analysis. The CysPAT approach was developed using a synthetic peptide, a standard protein and further the method was applied to detect total Cys residues from HeLa cells lysate with very high specificity and enrichment efficiency. The strategy was subsequently applied to simultaneously enrich cysteine-containing peptides and phosphorylated peptides from SILAC (stable isotope labeling by amino acids in cell culture) HeLa cells subject to epidermal growth factor (EGF) stimulation, which result in high enrichment specificity for both PTMs.

In this research, a substantial modulation of reversibly modified cysteine residues, presumably caused by the increase in hydrogen peroxide production after EGFR stimulation, was demonstrated. Additionally, the regulation of cysteine residues in numerous enzymes that are associated with dynamic PTMs have been observed. Most of the regulated cysteine sites have never been associated with EGF signaling before, and this could open up various new ideas to manipulate this pathway, which could be very important in a range of diseases [70].

In the proposed CysPAT strategy, almost complete labeling of the cysteine-containing peptides has been observed with minor detectable side reactions. Furthermore, high enrichment efficiency when using TiO_2_ with no side effects on peptide solubility, have been observed. Moreover, this approach makes possible the analysis and characterization of different PTMs such as phosphopeptides and sialylated glycopeptides, which have an affinity for TiO_2_ [70].

## 3. Methods of Thiopeptides Enrichment on Solid Supports

### 3.1. Michael Addition

Maleimide derivatives have been successfully used for the modification of thiol groups due to their high specificity, reactivity, stability of the resulting thioether product and the absence of by-products [71]. This specific reaction has been used, for example, to derivatize biomolecules and to conjugate various compounds [72]. Moreover, the use of maleimide derivatives for immobilization through monolayers on various glass, metallic and polymer surfaces has been presented [73,74]. Additionally, maleimide functionalized thiol-reactive semi-telechelic and telechelic polystyrenes were also successfully applied in the bio-immobilization of cysteine-containing peptide as presented by Tolstyka and co-workers [75].

Incorporation of maleimide into a polymer, such as a polyethylene glycol-based hydrogel, allows for relatively easy and effective functionalization of the material by thiol-containing compounds, such as selected fluorescent dyes, or the immobilization of biological compounds, e.g., proteins or peptides, containing cysteine residues [71]. Park and colleagues proposed the synthesis of a new hydrogel functionalized with maleimide by photopolymerization at room temperature. The first step was to attach a PEG-DA linker (PEG diacrylate) and a furan-coated maleimide monomer (FuMaMa) to the PEGMEMA comonomer in the presence of UV radiation. Then, the maleimide protecting group was removed by using a retro-Diels-Alder reaction in the presence of toluene and high temperature. The polymer prepared in this way was used, among others, in the process of biotinylation and then immobilization of the FITC-streptavidin protein (non-covalently binding biotin like avidin [76] with the attached FITC fluorescein isothiocyanate molecule), containing a cysteine residue.

**Figure 14:**
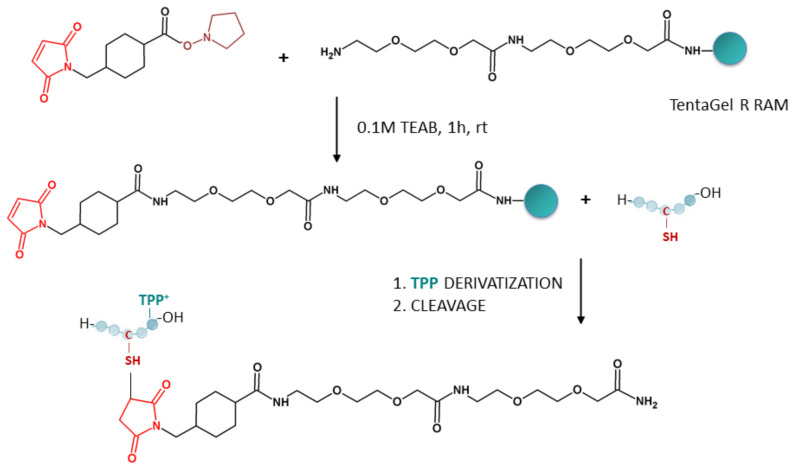
Schematic representation of the preparation of TentaGel R RAM resin, capturing of cysteine-containing peptides and their derivatization by fixed charge tag. *N*,*N*,*N*-triethylammonium bicarbonate, TPP—2,4,6-triphenylpyrylium salt [77].

Recently, we have developed a method of capturing cysteine-containing peptides resulting from the digestion of proteins with trypsin. The strategy used the commercially available TantaGel R RAM resin, which was modified with a spacer (9-aza-3,6,12,15-tetraoxa-10-na-heptadecanoic acid), increasing the distance between the solid support and the maleimide reactive group to cysteine thiol group (thio-Michael active site) [77]. This research used a model peptide, which was a trypsin fragment of podocin (a potential biomarker of preeclampsia). The model peptide was incubated with the modified solid support in 0.1 M TEAB (*N*,*N*,*N*-triethylammonium bicarbonate) at room temperature. In order to increase the ionization efficiency, the captured peptide containing *C*-terminal lysine was modified on the resin with a quaternary ammonium tag (2,4,6-triphenylpyrylium salt) (Figure 14), according to the method previously described by us [48,74]. Derivatization increased the intensity of the signals corresponding to the final products by more than 100 times. During the ESI-MS analysis, only signals corresponding to the captured and derivatized peptide were observed, which confirmed the effectiveness of the capture performed. Moreover, the developed method was tested on a more complex sample—podocin trypsin hydrolysate, and its effectiveness was also confirmed using such a system [77]. 

### 3.2. Thiol-Disulfide Exchange

Activated Thiol-Sepharose (agarose-(glutathione-2-pyridyl disulfide) conjugate, ATS) is a commercially available resin with an activated disulfide structure that reacts efficiently with –SH groups to form a disulfide bridge [78]. 

In 1975 Egorov and co-workers [79] conducted research using Activated Thiol-Sepharose to immobilize cysteine-containing proteins by creating a disulfide bridge between substrates. Activated Thiol-Sepharose was washed with an excess of coupling buffer (pH 8) and suspended in a 5-fold volume of buffer. Protein was added to a 10-fold molar excess of active groups. The reaction was run at room temperature. The captured proteins were proteolytically digested, washed, and then eluted with a reducing agent. This strategy was applied to the major parvalbumin (a protein made of 108 amino acid residues and only one cysteine residue), to human serum ferroxidase (1065 resides and 3 cysteines), and to mercaptalbumin from bovine serum (565 residues and one cysteine). With this technique, cysteine-containing parts of proteins can be isolated and studied. Similarly, disulfide bridges can also be isolated by the described method, after alkylation of the cysteinyl thiol groups and subsequent reduction. Moreover, it should be possible to extend the solid-phase approach to several of the existing specific and reversible modification reactions of amino acid side chains if these reactions are used for designing new appropriate absorbents.

Another use of ATS was demonstrated in 1998 by Caldas et al. [80]. The proposed strategy was a column format for purification of catalytically essential elongation cysteines of Escherichia coli. Applicability of ATS was studied for example in 2004 by Lee and co-workers [81] using disulfide-bridged proteins of plants. In 2005 Lee et al. [82] used ATS to enrich thiol-containing proteins from human epidermal cell cultures treated with arsenite. 

**Figure 15:**
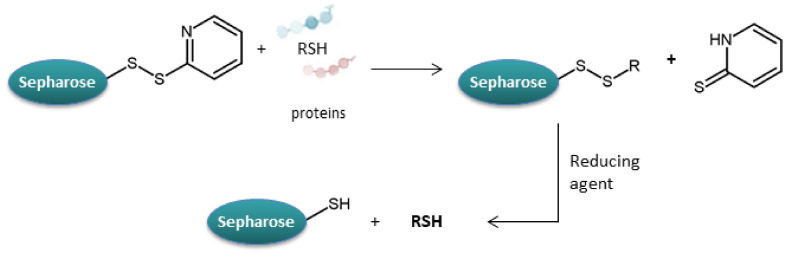
Schematic representation of cysteine-containing peptide enrichment by ATS [85].

In 2010 Hu et al. [83] studied the potential of batch-based ATS selection of –SH and –S–S-containing proteins for redox proteomics in DH5-Alpha Component E. coli by comparing selected subproteomes from control cells with those grown in the presence of the pro-oxidant menadione. A batch protocol facilitates parallel enrichment of low-abundance proteins in multiple matched samples. Activated Thiol-Sepharose facilitates selection of thiol-containing proteins. The procedure involved unfolding proteins with urea, then incubating proteins with ATS in binding buffer solution (Tris-HCl, pH = 7.5, NaCl, EDTA). ATS beads swelled, efficiently absorbing the protein solution. Then the sample was washed with binding buffer eight times and supernatants were discarded. The last step involved incubation with binding buffer containing DTT, centrifugation and collection of supernatant. In the case of proteins containing disulfide bridges, they were first treated with *N*-ethylmaleimide (NEM)—thiol-specific reagent, widely used to block free thiols, which provided a strategy for selective reduction of disulfide bridges [84]. Then excess NEM was removed, and proteins were treated with DTT (4 °C, 1 h) to reduce disulfide bridges to free thiols (Figure 15). Finally, the excess DTT was removed and proteins were selected on ATS [85]. 

The captured proteins were enzymatically digested and analyzed. 183 thiol-containing proteins were identified, and more than 90% of the proteins identified contained at least one cysteine residue. ATS facilitates the rapid and quantitative selection of key proteins containing thiol- or disulfide-containing subproteomes, essential for key biological processes (e.g., translation, metabolism, oxidative stress). Comparing this strategy with other identification methods, it was found that the identified subproteomes coincide with those identified in previous studies [85,86,87]. The thiol-containing proteins were either present in the control cells only or were present in both the control and menadione-treated cells. In several cases, proteins only appear as disulfides in menadione treated cells due to disulfide formation in response to oxidative stress. Consequently, ATS is suitable for probing thiol oxidation and thus contributes to redox proteomics [80].

**Figure 16:**
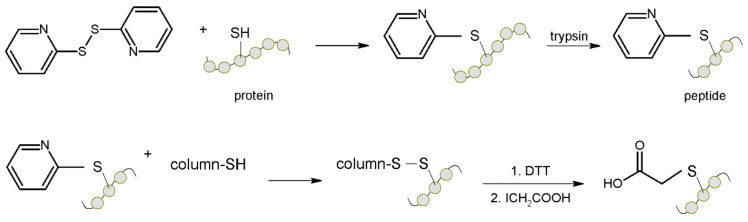
Schematic representation of capture of cysteine-containing peptides from trypsin digestion using thiol-disulfide exchange covalent chromatography. DTT—dithiothreitol [88].

In 2001 Wang et al. [88] developed a procedure for the capture of cysteine-containing peptides from trypsin digestion using thiol-disulfide exchange covalent chromatography. The procedure consisted of disrupting the disulfide bonds with 2,2′-dipyridyl disulfide, digestion with trypsin and acylation with succinic anhydride. The cysteine-containing peptides were then captured on a Thiopropyl Sepharose resin by thiol-disulfide exchange. The peptides were then released with a dithiothreitol solution containing (ethylenedinitrilo)tetraacetic acid disodium salt (pH 7.5) (Figure 16), alkylated with iodoacetic acid and fractionated by reverse phase liquid chromatography (RPLC). The collected fractions were analyzed by MS analysis. The obtained test results showed that the capture of cysteine-containing peptides reduces the complexity of the sample. Combining this technique with isotopic labeling can further expand and facilitate quantification and determination of protein concentration changes between samples. Using this strategy, upregulated E. coli proteins were identified. 

In 2002, Wang et al. [89] described the procedure of capturing cysteine and histidine containing peptides from the digestion products of cell lysates. First, the cysteine-containing peptides were captured by chromatography. Then, after the cysteine-containing peptides were released from the column, the histidine-containing peptides were captured by passage through an immobilized metal (Cu) affinity chromatography column. The quantification of the captured peptides was also performed. For this purpose, labeling of control and experimental samples with isotopically different forms of succinic anhydride was performed, both samples were mixed, and fractionation of labeled peptides was performed by RPLC. Then the MS analysis was performed. The results of these studies indicated that by capturing peptides containing both cysteine and histidine, the complexity of the samples could be significantly reduced (up to 95%). The performed studies allowed for the identification and quantification of the upregulated proteins from plasmid bearing *Escherichia coli*. 

**Figure 17:**
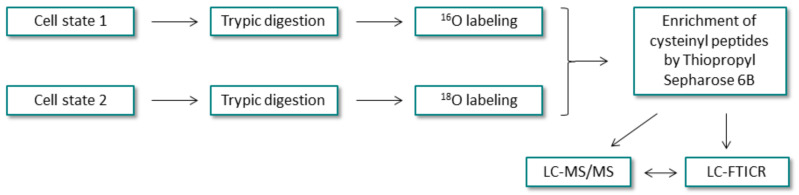
Scheme representing the strategy developed by Liu and co-workers [33].

In 2004, Liu et al. [33] proposed another method for the quantitative enrichment of cysteine-containing peptides to achieve higher yield, greater dynamic range and higher throughput in quantitative proteomics. This strategy used two mixtures of proteins representing different cellular states. These samples were separately digested by trypsin and then labeled by trypsin-catalyzed oxygen isotope exchange in ^16^O and ^18^O enriched water, respectively (Figure 17). The samples were then combined and the cysteine containing peptides were selectively captured by the Thiopropyl Sepharose 6B resin. Enriched peptides were subjected to LC-MS/MS analysis and LC-Fourier ion cyclotron resonance analysis with MS ion transformation (LC-FTICR). Proteome profiling of native and in vitro differentiated human mammary epithelial cells using the developed strategy allowed the identification of 603 proteins in a single LC-FTICR analysis. 

The enrichment of cysteine-containing peptides by Thiopropyl Sepharose resin is highly efficient and can easily be automated. The thiopeptide capturing reaction has no side products. In addition, the lack of an introduced isotope labeled tag, eliminates the problem of generation of fragmentation ions from the tags (e.g., as experienced by ICAT and ICAT-like reagents) during CID [90]. In addition, isotope labeling after trypsin digestion resulted in the introduction of two ^18^O atoms in almost all trypsin peptides, which is a good basis for an accurate quantification [33].

**Figure 18:**
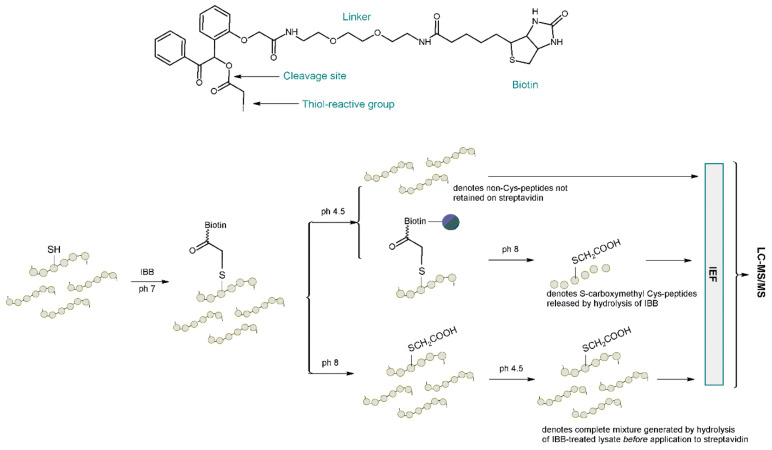
Chemical structure of biotinylating tag (IBB) and schematic representation of the approach for evaluation of IBB and streptavidin capture of cysteine-containing peptides [1].

The research of Lin et al. [1] extends the findings of Liu [33] and Wang [85,86] by quantifying the enhanced detection of low abundance proteins in yeast (Saccharomyces cerevisiae) and extending this quantification to the proteome of human colon carcinoma RKO cells. Lin et al. in 2010 used a biotinylating tag *N*-(2-(2-(2-(2-(3-(1-hydroxy-2-oxo-2-phenylethyl)phenoxy)acetamido)ethoxy)-ethoxy)ethyl)-5-(2-oxohexahydro-1H-thieno[3,4-d]imidazol-4-yl)pentanamide (IBB) to capture cysteine-containing peptides reacting selectively with thiols. The procedure consisted of digesting proteins with trypsin and then treating the digestion products with the proposed tag. The next step was the capture of streptavidin and hydrolysis in a mild alkaline environment which released the cysteine containing peptides with a residual carboxymethyl tag, followed by isoelectric focusing (IEF) fractionation followed by LC-MS/MS analysis (Figure 18). 

IBB-based fractionation improved the detection of cysteine-containing proteins in direct proportion to their cysteine content. A 2–8 fold increase in the enrichment degree of cysteine-containing peptides was observed, and in some cases up to a 20 fold increase in the enrichment degree was observed. An important part of this research was the use of a previously quantified [91] reference proteome from yeast, that serves as a benchmark for comparison of spectral data obtained during LC-MS/MS analyses [92]. This provided an appropriate method for validation of the cysteine-containing peptide enrichment approach that was developed. The resulting method made it possible to increase the detection of cysteine-containing proteins, which was particularly noticeable in the case of lower abundance cysteine-containing proteins in the sample. This was probably due to the chemoselectivity rather than the simplification of the peptide mixture by fractionation.

**Figure 19:**
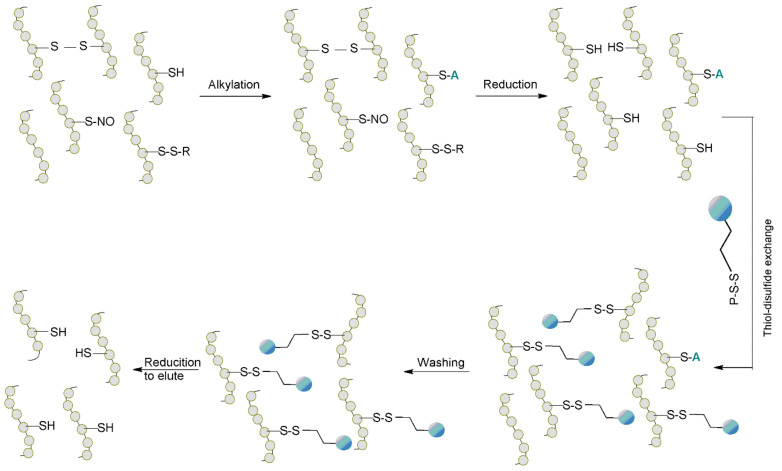
A schematic representation of the thiol-disulfide exchange chromatography method [93].

In 2013, Paulech et al. [93] developed an enrichment method that relied on specific alkylation of free cysteine followed by thiol-based reduction to convert the reversibly oxidized cysteine to free thiols. The cysteine-containing peptides were then captured by the functionalized resin (Thiopropyl Sepharose 6B) via thiol-disulfide exchange. Non-covalently bound proteins or peptides were washed out, and then the disulfide bridges were reduced to elute the cysteine-containing peptides (Figure 19). The chromatographic conditions were optimized to provide increased specificity by removing non-covalent interactions. Mass spectrometry analysis determined that the developed method was highly efficient; the reaction with the resin proceeded in an equimolar ratio and was repeatable, with linear elution of the peptides. These features make this method suitable for quantifying relative fold estimates.

The described strategy was applied to a complex protein lysate prepared from rat cardiac muscle tissue, and 6559 unique peptides containing cysteine from 2694 proteins were identified, demonstrating successful protein enrichment. Analysis of amino acid sequence features indicated a preference for acid residues and increased hydrophilicity in the regions immediately upstream or downstream of the reactive Cys [90].

In 2014, Guo et al. [94] described the procedure of enrichment of peptides containing reversible cysteine modifications. Free thiol groups were blocked with NEM, and then cysteine side chains were reduced with specific reducing agents that react selectively with each type of modification (ascorbate in the case of S-nitrosylation, S-glutonylation of glutaredoxin, hydroxylamine in the case of S-acylation and DTT in the case of complete reduction (pre-processing)). Capture was performed on tissue samples and cell lysates using Thiopropyl Sepharose 6B resin and a thiol disulfide exchange reaction. The proteins were then digested with trypsin, followed by isotope labeling to facilitate analysis by LC-MS/MS. In a successful enrichment experiment performed according to the developed procedure, >95% of the final identified peptides should be cysteine containing, resulting from the high specificity of this approach.

**Figure 20:**
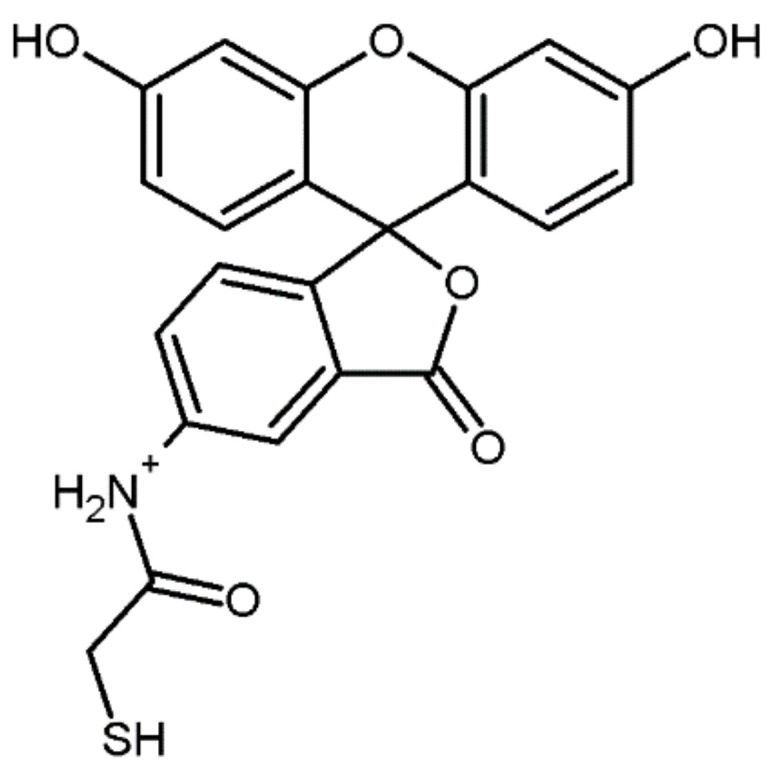
Chemical structure of 5-iodoacetamido-fluorescein [97].

Fluorescent labeling in proteomics is useful in tracking and quantifying target proteins during sample preparation or chromatographic processes. In 2008 Chen et al. [95] redeveloped a method of labeling cysteine residues using a fluorophore (a derivative of fluorescein). Such visible dyes have been shown to have many unique properties, including a unique reporter ion containing a dye moiety due to collision-induced dissociation and high affinity for multicarboxylic functional groups, which may be useful for increasing selectivity in MS-based proteomics. The study used 5-iodoacetamido-fluorescein (Figure 20) to selectively react with a sulfhydryl group. The labeling was performed on the intact protein ovalbumin, bovine serum albumin and MCF-7 cells (human breast cancer cell line with estrogen, progesterone and glucocorticoid receptors [96]). Proteins were digested with trypsin and then analyzed by nanoLC-ESI-Q-TOF or MALDI-TOF. As a result, spectra similar to unlabeled or derivatized proteins with iodoacetamide were obtained, and a strong reporter ion containing a fluorescein moiety was observed during fragmentation. 

Using a reporter ion precursor scan, the cysteinyl protein ovomucoid was identified as an impurity in the ovalbumin sample. Additionally, the combination of isotope labeling of a fluorescein-derivatized peptide facilitates selective enrichment, identification and quantification. The presented method can be used to capture peptides or proteins containing other amino acids by using various fluorescein dyes containing different functional groups.

**Figure 21:**
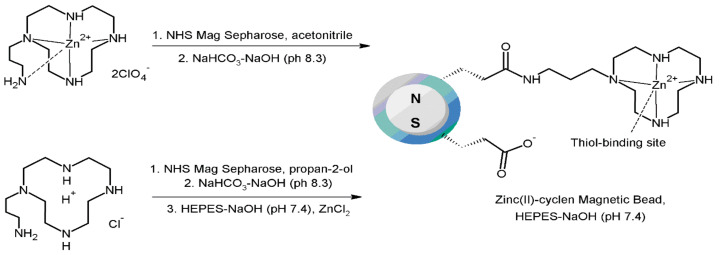
Schematic representation of the zinc(II)-cyclen magnetic beads preparation. NHS—*N*-hydroxysuccinimide, HEPES—4-(2-hydroxyethyl)-1-piperazineethanesulfonic acid [97].

In 2014 Fujioka et al. [97] developed a method for enrichment of thiol-containing biomolecules based on magnetic-bead technology. The thiol-binding site on the bead is a mononuclear complex of zinc(II) with 1,4,7,10-tetraazacyclododecane (cyclen), Figure 21. The binding site is linked to a hydrophilic cross-linked agarose coating, on a particle that contains a magnetic core. Separation and capture of thiol-containing molecules was performed in several aqueous buffers (neutral to mildly acid pH). The designed magnetic bead can be used multiple times (at least 15 times) without affecting their ability to bind thiols. 

The presented method was applied in the separation of cysteine-containing peptides (Cys-conjugated β-amyloid(1–12) dodecapeptide and rat MOG(91–108)peptide) from an excess of two reference peptides (methionine-containing pentapeptide (Met-enkephalin) and β-amyloid-binding peptide consisting of 20 amino acid residues). Both cysteine-containing peptides were efficiently separated from the reference peptides, the recoveries in one fraction were 67% and 52%, respectively. The remaining Cys-containing peptides on the beads were eluted, resulting in total recoveries of more than 98%. The methods allowed for the complete elimination of the reference peptides. Subsequently, they examined a more-complex system containing three cysteine-peptides (Cys-conjugated β-amyloid(1–12)dodecapeptide, rat MOG(91–108)peptide, and cysteine-containing enolase peptide (Ile-Gly-Leu-Asp-Cys-Ala-Ser-Ser-Glu-Phe-Phe-Lys)) and a tryptic digest of β-casein (protein without cysteine residue). The three cysteine peptides were preferentially collected in one fraction (recoveries of 51, 64, and 62% respectively). The presented separation procedure is efficient, the separation time is short, and involves the use of neutral to mildly acid buffers, which makes the proposed method suitable for general biological research [94].

Another cysteine modification can be PEGylation, covalent bonding of polyethylene glycol through thiol groups of cysteine residues, or ℇ-amino groups of lysine residues, contained in proteins of therapeutic importance, which is intended to enrich these compounds [98,99,100]. The reaction can be carried out in an ultrafiltration reactor using the FASP [101,102] which includes such steps as efficient digestion of proteins with an appropriate enzyme and allows for thorough mixing of the reagents involved in the subsequent stages of the reaction [103]. It is used, for the capture and selective elution of lectin-binding peptides, antibodies and for the analysis of complex protein complexes purified by affinity chromatography, amongst other purposes [103,104]. 

**Figure 22:**
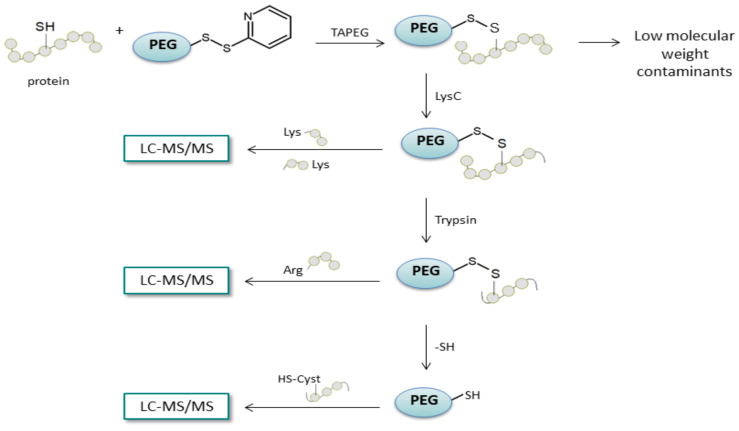
Schematic representation of enrichment of cysteine-containing peptides, by the FASAP method. TAPEG—thiol-activated polyethylene glycol [105].

In 2015 Wiśniewski and Pruś [105] described a proteomic reactor-based homogeneous phase enrichment of cysteine-containing peptides in a filtered sample preparation (FASP) format. In this method, the disulfide bridges were reduced and then derivatized with thiol-activated polyethylene glycol (TAPEG). The captured protein was digested with LysC endoproteinase and trypsin, which allowed the isolation of two fractions. Subsequently, reduction of the disulfide bridges formed between the PEG and the cysteine residue was performed, and another peptide fraction was collected (Figure 22). Additionally, the presented strategy can be extended with isotope labeling in order to perform accurate proteomic analysis.

Analysis of whole cell lysates of red muscle fibers, liver and brain of mice, as well as CaCo-2 cells, using this approach, allowed the identification of 4200, 5800, 6900 and 7900 proteins, which is 10–30% more than were identified using two-step digestion without isolating cysteine-containing peptides. It has been shown that high molecular weight, functionalized PEG can be used for enrichment in the FASP format. High molecular weight PEG can be functionalized in a number of ways, making it a potentially appropriate biochemical tool for use in the micro-range desired for cysteine redox proteomics [102]. In addition, the strategy was applied to peptide capture by antibodies and lectin, showing the utility of method [106,107].

## 4. Discussion

The analysis of peptides and proteins present in samples of biological origin is difficult due to the complicated composition of the tested mixture and the low sensitivity of the analytical methods, preventing the analysis of trace amounts [1]. One of the most frequently used analytical methods in peptide and protein studies is liquid chromatography coupled with mass spectrometry (LC-MS) and tandem mass spectrometry (MS/MS) [108]. However, the low ionization efficiency of some compounds may make them difficult to identify [34]. Additionally, in order to analyze the peptides formed during protein hydrolysis, they must first be separated from the test mixture, which can also be a challenge. Therefore, methods are needed that allow for the selective enrichment of specific peptides, including thiopeptides, by labeling of appropriate functional groups, and by increasing the ionization efficiency of the obtained analytes, to enable their ultra-sensitive analysis [30,37].

Cysteine, due to its common occurrence in tryptic hydrolysates and the high reactivity of the thiol group [1], is an attractive compound to modify in order to improve the detection and increase the sensitivity of analytical methods. In this review, we presented various strategies to capture and increase the ionization efficiency of cysteine-containing peptides.

The developed strategies for the enrichment of cysteine-containing peptides are mainly based on selective alkylation of cysteine thiols by biotin [73], maleimide [21], or bromomaleimide [25], thiol-disulfide chemistry (mainly by using thiopropylsepharose [76,78]) and charge derivatization by a quaternary amine tag [20]. Additionally, an enrichment method using nanoparticles has been developed [29]. The methods of enrichment of cysteine-containing peptides can be divided into enrichment in solution and enrichment on a solid support.

The Michael addition reaction occurs with the α, β-unsaturated carbonyl moiety containing compounds, and a thiol-ene is formed [16]. Maleimide and its derivatives have been used extensively to modify cysteine residues [5,25]. The advantage of cysteine modification by maleimide is its high selectivity and irreversibility [22,109]. However, in comparison to, for example methanethiosulfonate, the reaction is slower and the reaction of cysteine with maleimides requires the monitoring of pH. A pH > 7 is desirable since the reaction is faster with the thiolate ion (S^−^). Although at pH > 8 maleimides will also react with deprotonated primary amines [106], which may lower the reaction efficiency and complicate data analysis. Therefore, in the case of the Michael addition-based reaction, the reaction conditions are very important. 

Other techniques of cysteine-containing peptide enrichment use thiol-disulfide exchange chemistry. The thiol-disulfide exchange reaction proceeds according to a S_N_2 reaction mechanism. Many developed methods used resin with an activated disulfide structure—Thiopropyl-Sepharose, and enrichment methods based on thiol-disulfide exchange with Ellman’s reagent [11], [33,76,77,78,79,80,91]. Activated Thiol-Sepharose facilitates the selection of thiol-containing proteins [80]. The enrichment of cysteine-containing peptides by Thiopropyl Sepharose resin is highly efficient and can be easily automated. The thiopeptide capturing reaction has no side products [87]. The resin assisted approach of cysteine-containing peptide enrichment is a specialized method to identify novel cysteine sites sensitive to reversible modifications or redox reactions [91]. This method requires the application of a disulfide containing a good leaving group (in the case of nucleophilic reaction of a thiol). The reaction is pH-dependent and proper development of reaction conditions is needed. 

The thiol-disulfide strategies were widely used and extensively modified. To enrich cysteine-containing peptides, nano-sized materials have been applied [30,31,32]. The application of nano-sized materials to proteomic research provides many immediate advantages, such as higher specificity, faster binding rates, higher surface-to-volume areas, and higher miscibility [29]. Application of superparamagnetic Fe_3_O_4_@SiO_2_ core–shell nanoparticles shows high efficiency cysteine-containing peptide capture, without contamination. The possible application of nanoparticles in cysteine-containing peptide enrichment combines peptide chemistry and materials science, and may lead to microdevices and nanomaterials. 

Another strategy developed to facilitate LC-MS analysis is charge derivatization for increasing ionization efficiency. We successfully used solid-support derivatization to analyze the OBOC (one-bead-one-compound) peptide library by MS/MS analysis of a trace amount of a compound obtained from single resin grains [46,47]. Other types of peptide ionization tags described previously by us [48] are the 2,4,6-trimethylpyryl and 2,4,6-triphenylpyryl salts, such as triphenylpyryl tetrafluoroborate (TPP), which allow the modification of the α-amino groups of glycine and alanine, as well as the ε-amino group of the lysine residue. *N*-(3-iodopropyl)-*N*, *N*, *N*-dimethyloctylammonium iodide serving as a derivatizing agent increases the detection threshold significantly [57]. Modifications of the cysteine residue have the advantage that they target the most nucleophilic functional group found in peptides, -SH, allowing the reaction to proceed selectively [56]. This approach leads to ionization efficiency enhancements and may facilitate data interpretation due to the formation of a characteristic series of fragment ions, or even reporter ions, the identification of which may clearly indicate the presence of the investigated biomarker.

Fluorescent cysteine residue labeling techniques have also been developed [92]. A derivative of fluorescein (5-iodoacetamido-fluorescein) has been used. Fluorescent labeling in proteomics is useful in tracking and quantifying target proteins during sample preparation or chromatographic processes. Furthermore, the combination of isotope labeling of a fluorescein-derivatized peptide facilitates selective enrichment, identification, and quantification. Moreover, a functionalized PEG can be used for the enrichment of cysteine-containing peptides [102]. PEG can be functionalized in a number of ways, making it a potentially appropriate biochemical tool for use in the micro-range desired in cysteine redox proteomics. This approach, together with the fixed-charge tag modification, significantly increase the sensitivity of detection, however, it requires some chemical transformations. 

It can be concluded that there is not one universal method of cysteine-containing peptide enrichment which could solve the problem of reaction selectivity, efficiency and sensitivity of detection. All of the above techniques have their advantages and disadvantages, but have made proteomic analyses much easier, and much more accurate.

The review of current methods used for the chemoselective modification of cysteine-containing peptides, based on their enrichment (catching) and chemical modification, offers a powerful tool for researchers working with complex biological mixtures. Targeting of cysteine-containing tryptic peptides in proteomes leads to a significant reduction of sample complexity while allowing high proteome coverage. The simplified proteomic strategies may allow access to low abundance proteins which may serve as biomarkers of certain diseases. This approach may improve diagnostic accuracy, allowing early diagnosis of diseases based on selected biomarker identification. Additionally, developed methods for cysteine-containing peptides and proteins may result in the formation of new therapeutics, including antibody-drug conjugates.

## 5. Conclusions

Investigation of peptide and protein biomarkers of cellular and tissue proteomes is limited by the complex nature of the samples. In this review, developed methods of cysteine-containing peptide enrichment for efficient and sensitive analysis, have been systematically summarized. The sulfhydryl group of cysteine side chains has strong nucleophilic properties, and is redox active, which means that cysteine can be easily modified. Numerous modifications can be used to capture cysteine-containing peptides and enable sensitive analysis. Modifications of cysteine residues containing a thiol group can be introduced in the Michael’s addition reaction. This is based on the reaction of an activated substrate, containing a double bond and a thiol group. The role of the activated substrate can be performed by maleimide or its derivatives. Several techniques to enrich cysteine-containing peptides based on thiol-disulfide exchange have been described, particularly those using Activated Thiol-Sepharose. Another strategy for the selective capture of thiopeptides used fixed charge tags (quaternary amines), which result in an increase in the ionization efficiency of the tested compounds. Described methods significantly facilitate analysis and may allow detection of peptides and proteins that had not been previously identified. However, they are not without their drawbacks. Therefore, future, research on the development of new methods of selective cysteine-containing peptide enrichment, should be focused on relevant applications, improving existing technologies to generate straightforward, efficient, easily implemented techniques that lead to the formation of stable conjugates, which facilitate analysis and increase the sensitivity of detection. 

## Data Availability

Not applicable.
